# Immunoglobulin G modulation of the melanocortin 4 receptor signaling in obesity and eating disorders

**DOI:** 10.1038/s41398-019-0422-9

**Published:** 2019-02-12

**Authors:** Nicolas Lucas, Romain Legrand, Christine Bôle-Feysot, Jonathan Breton, Moïse Coëffier, Kirsti Akkermann, Anu Järv, Jaanus Harro, Pierre Déchelotte, Sergueï O. Fetissov

**Affiliations:** 1 Inserm UMR1073, Nutrition, Gut and Brain Laboratory, 76183 Rouen, France; 2 Institute for Research and Innovation in Biomedicine (IRIB), University of Rouen Normandy, 76000 Rouen, France; 3 Division of Clinical Psychology, Department of Psychology, University of Tartu, Näituse 2, 50409 Tartu, Estonia; 4 Tartu University Clinics, Psychiatric Hospital, University of Tartu, Ludvig Puusepa 1a, 50406 Tartu, Estonia; 5 Division of Neuropsychopharmacology, Department of Psychology, University of Tartu, Ravila 14A, 50411 Tartu, Estonia; 6 Rouen University Hospital, CHU Charles Nicolle, 76183 Rouen, France; 7 Present Address: Inserm UMR1239, 25 rue Licien Tésniere, 76130 Mont-Saint-Aignan, France

## Abstract

Melanocortin 4 receptor (MC4R) plays a key role in regulation of appetite activated by its main ligand α-melanocyte-stimulating hormone (α-MSH) in both central and peripheral targets. α-MSH also binds to circulating immunoglobulins (Igs) but the functional significance of such immune complexes (ICs) in MC4R signaling in normal and pathological conditions of altered appetite has remained unknown. To address this question, we analyzed plasma levels, affinity kinetics, and binding epitopes of α-MSH-reactive IgG extracted from plasma samples of female patients with hyperphagic obesity, anorexia nervosa, bulimia nervosa, binge-eating disorder, and healthy controls. Ability of α-MSH/IgG IC to bind and activate human MC4R were studied in vitro and to influence feeding behavior in vivo in rodents. We found that α-MSH-reactive IgG were low in obese but increased in anorectic and bulimic patients and displayed different epitope and kinetics of IC formation. Importantly, while α-MSH/IgG IC from all subjects were binding and activating MC4R, the receptor binding affinity was decreased in obesity. Additionally, α-MSH/IgG IC had lower MC4R-mediated cAMP activation threshold as compared with α-MSH alone in all but not obese subjects. Furthermore, the cellular internalization rate of α-MSH/IgG IC by MC4R-expressing cells was decreased in obese but increased in patients with anorexia nervosa. Moreover, IgG from obese patients prevented central anorexigenic effect of α-MSH. These findings reveal that MC4R is physiologically activated by IC formed by α-MSH/IgG and that different levels and molecular properties of α-MSH-reactive IgG underlie biological activity of such IC relevant to altered appetite in obesity and eating disorders.

## Introduction

Molecular mechanisms underlying altered appetite in common obesity and in eating disorders (EDs) need further elucidation. Activation of the melanocortin 4 receptor (MC4R) by melanocortin peptides such as α-melanocyte-stimulating hormone (α-MSH) is a critical molecular pathway regulating feeding behavior and energy balance by inducing satiety and increasing energy expenditure^1–4^. Indeed, inactivation of either α-MSH precursor proopiomelanocortin or of MC4R lead inevitably to hyperphagia, increased preference for fat food, and obesity in genetically modified rodents and may underlie about 2% of genetic causes of obesity in humans^2,5–8^. Target sites of MC4R signaling include both the central and peripheral nervous systems as well as the gut^9–11^. However, no clear genetic alterations, including of genes involved in MC4R signaling, have been detected in the major forms of obesity and ED^12^.

Immunoglobulins (Igs) reactive with α-MSH are ubiquitously present in humans and rodents and their production is linked to the presence of homologous antigens synthesized by gut bacteria^13–16^. Intriguingly, plasma levels of α-MSH-reactive IgG correlate with disease-characteristic psychopathological traits in ED patients, but the underlying molecular mechanisms have remained unknown^17^. The ubiquitous presence of α-MSH-reactive IgG in the circulation suggests that they may constitutively modulate α-MSH signaling by forming immune complexes (ICs), but whether this influences MC4R activation is unknown. A putative functional effect of α-MSH/IgG IC may contribute to the individual variability of α-MSH MC4R activation relevant to conditions of altered feeding behavior in ED and in hyperphagic obesity. Such IgG-modulatory mechanism may complement other non-genetic mechanisms affecting α-MSH signaling through MC4R, including α-MSH degradation by prolylcarboxypeptidase, functional antagonisms by agouti-related protein (AgRP), cholesterol-dependent MC4R endocytosis, etc^18–21^.

In the present study, we addressed the question of the possible functional role of α-MSH-reactive IgG in MC4R signaling and further analyzed whether this role is altered in patients with hyperphagic obesity or ED, including anorexia nervosa (AN), bulimia nervosa (BN), and binge eating disorder (BED). For this purpose, we analyzed the affinity kinetics of α-MSH/IgG IC formation in patients and controls (Ctrl), screened the epitopes, and determined whether α-MSH/IgG IC may activate human MC4R in vitro (receptor binding and internalization and cellular cyclic adenosine monophosphate (cAMP) production). Finally, we evaluated in rats the effects of central administration of α-MSH/IgG IC on feeding behavior as well as the relevance of plasmatic Ig to α-MSH anorexigenic effects in transgenic Ig-deficient mice.

## Patients, materials, and methods

### Plasma samples from patients and controls

Plasma samples were obtained from obese (OB) female patients all reporting hyperphagia without BED (body mass index [BMI], mean ± standard deviation, 37.51 ± 5.0 kg/m^−2^, age 47.2 ± 16.3 years, *n* = 17), from female patients with restrictive AN (BMI, 15.01 ± 1.99 kg/m^−2^, age 18.6 ± 4.9 years, *n* = 28), BN (BMI, 21.71 ± 3.67 kg/m^−2^, age 22.4 ± 6.8 years, *n* = 34), and BED (BMI 33.34 ± 8.16 kg/m^−2^, age 30.6 ± 11.6 years, *n* = 14). Plasma samples from healthy female participants were used as controls (BMI, 22.60 ± 3.77 kg/ m^−2^, age 25.8 ± 8.7 years, *n* = 65). ED were diagnosed by a psychiatrist and a clinical psychologist according to the Diagnostic and Statistical Manual of Mental Disorder IV^22^. Sample sizes were selected based on previous experiments on analyzing IgG properties in OB and AN patients^23^. Venous blood samples were taken in the morning before breakfast and centrifuged at 3000 rpm for 20 min at 4 °C to collect plasma. Samples were conserved at −80 °C. The studies were approved by the Institutional Ethical Committees and all patients gave their informed consent for study participation. It is of note that plasma samples from OB patients were obtained in Rouen University clinic (France) while plasma samples of ED patient and controls were from Tartu University clinic (Estonia). While some differences in plasma sampling procedure may be present, their potential influence on the results were minimized by studying IgG extracted from all plasma samples using the same protocol in the same laboratory. We cannot exclude whether differences in patient’s geographic location and age as well as potential comorbidities may have influenced the data.

### Total IgG and α-MSH-reactive IgG purification

Total IgG were purified from plasma using the MelonGel® Purification Kit (Life Technologies, Carlsbad, CA, USA) as described in supplementary methods. The same stock solution of IgG from each patient and control was used throughout the study. Based on affinity kinetics analysis, we selected IgG for pooling from each study group (OB, *n* = 10; AN, *n* = 10; BN, *n* = 8; BED, *n* = 7, and controls *n* = 10), with most differences from the mean values of small kd in the controls. Pooled total IgG were further purified using α-MSH-linked UltraLink® Biosupport (Thermo Fisher Scientific, Waltham, MA, USA) as described in supplementary methods. α-MSH peptide for this and other experiments was purchased from Bachem AG (Bubendorf, Switzerland). Purified IgG were lyophilized, resuspended in phosphate-buffered saline (PBS), and conserved at −30 °C.

### Affinity kinetics of IgG for α-MSH and of α-MSH/anti-α-MSH IgG IC for the MC4R

Affinity kinetics of plasma-extracted IgG for α-MSH and of α-MSH/IgG IC for MC4R were analyzed by biospecific interaction assay based on surface plasmon resonance (SPR) using BIAcore 1000 instrument (GE Healthcare), with coating of α-MSH peptide on the biosensor surface according to previously published studies and a protocol^15,23,24^. A cell line of human embryonic kidney (HEK) 293 cells stably expressing human MC4R (hMC4R) with green fluorescent protein (GFP) in its promoter region was purchased from AMS Biotechnology (Abingdon, UK). Cells were coated on the biosensor surface as described in supplementary methods. Affinity kinetic data were analyzed using the BiaEvaluation 4.1.1 program (GE Healthcare) and fitted with the Langmuir’s 1:1 model after blank value subtraction.

### Confocal microscopy

MC4R-expressing HEK 293 cells (AMS Biotechnology, Abingdon, UK) were cultured in glass bottom Petri dishes (MatTek, ≈250,000 cells/dish). Purified anti-α-MSH IgG of each patient and control groups (2 mg/mL in PBS) were conjugated with DyLight® 550 using the Lightning-Link® Rapid Conjugation Kit (Innova Biosciences, Cambridge, UK) and then incubated with 200 mM of α-MSH peptide overnight at 4 °C. After incubation for 30 min at 37 °C, cells were fixed in 4% paraformaldehyde and observed on an inverted confocal laser scanning microscope TCS SP2 AOBS-DMIRE2 (Leica, Wetzlar, Germany). Pictures were acquired with ×45 and ×60 oil-immersion objectives at the optical planes where cell nuclei were the most voluminous. Data were analyzed using the Leica Confocal Software (Leica) and red DyLight® 550 spots in GFP+ cells were quantified (*n* = 50 for each condition).

### In vitro cAMP assay

cAMP production by MC4R-expressing HEK 293 cells was measured using the bioluminescence assay cAMP-Glo^TM^ Max Assay Kit (Promega, Madison, WI, USA) as previously described^25^. α-MSH peptide was preincubated overnight at 4 °C before the experiment with pooled purified IgG diluted in PBS. To confirm the specificity of MC4R-dependent cAMP release, MC4R + HEK 293 cells were preincubated with the MC4R reverse agonist AgRP (100 nM, Bachem). To confirm the specificity of α-MSH-reactive IgG effects on MC4R signaling, α-MSH was preincubated overnight at 4 °C with IgG depleted from α-MSH-reactive IgG (*n* = 3/group) or with affinity-purified α-MSH-reactive IgG (*n* = 6/group). For details, see supplementary methods.

### α-MSH-reactive IgG epitope mapping

α-MSH IgG autoAbs epitope mapping was performed using enzyme-linked immunosorbent assay following adsorption of IgG by tetrapeptide fragments spanning the α-MSH sequence as described in supplementary methods.

### Brain injections of α-MSH IgG IC in rats

Animal care and experimentation were in accordance with guidelines established by the National Institutes of Health, French and European Community regulations (Official Journal of the European Community L 358, 18/12/1986) and the study was approved by a Regional Ethical Committee (Rouen, France). Sample sizes were selected based on previous experiments^26^. Non-randomized male Sprague-Dawley rats were anesthetized by ketamine (75 mg/kg, Virbac, Carros, France)/xylazine (5 mg/kg, Bayer, Leverkusen, France) solution (3:1 vol., 0.1 mL/100 g, intraperitoneal (IP)) and were stereotaxically implanted with a 9-mm guide cannula (PlasticsOne, Roanoke, VA, USA) into the paraventricular nucleus (PVN). Upon awakening, rats were individually maintained in metabolic cages (Techniplast, Louviers, France) for 5–6 days. At day 6, 12-h fasted rats were injected at the beginning of the dark phase with 2 µL of α-MSH peptide (2.5 mg/mL) alone (*n* = 11) or preincubated with 0.82 mg/mL of IgG from AN (*n* = 5), BN (*n* = 5), BED (*n* = 6), OB (*n* = 6), or Ctrl (*n* = 4) subjects in artificial cerebrospinal fluid (aCSF; Phymep, Paris, France). A control group (*n* = 5) was injected with 2 µL of aCSF. Food intake was measured 30 min and 2 h after injection.

### IP administration of α-MSH in immunodeficient Rag^−/−^ mice

Rag^−/−^ mice (*n* = 6), bred in a specific-pathogen-free area, were generously provided by Professor Olivier Boyer and Mrs Laetitia Jean, Inserm 1234, Rouen. Rag^−/−^ and C57Bl/6 male mice were individually housed in BioDaq® cages (Research Diets Inc., New Brunswick, NJ, USA) for 7 days, and the basal food intake of each animal was measured during the first 4 h of the dark phase. At day 8, IP injections of α-MSH (100 µg/kg, Bachem) were performed at the beginning of the dark phase. Individual food intake after injection was compared to the basal food intake of the pretreatment period.

### Statistical analysis

Data were analyzed and graphs plotted using GraphPad Prism 5.02 (GraphPad Software Inc., San Diego, CA, USA). All results were expressed as mean ± standard error of mean (s.e.m.), unless specified; for all tests, *p* < 0.05 was considered statistically significant.

## Results

### α-MSH-reactive IgG form IC with different affinity kinetics in OB and ED patients

**Fig. 1:**
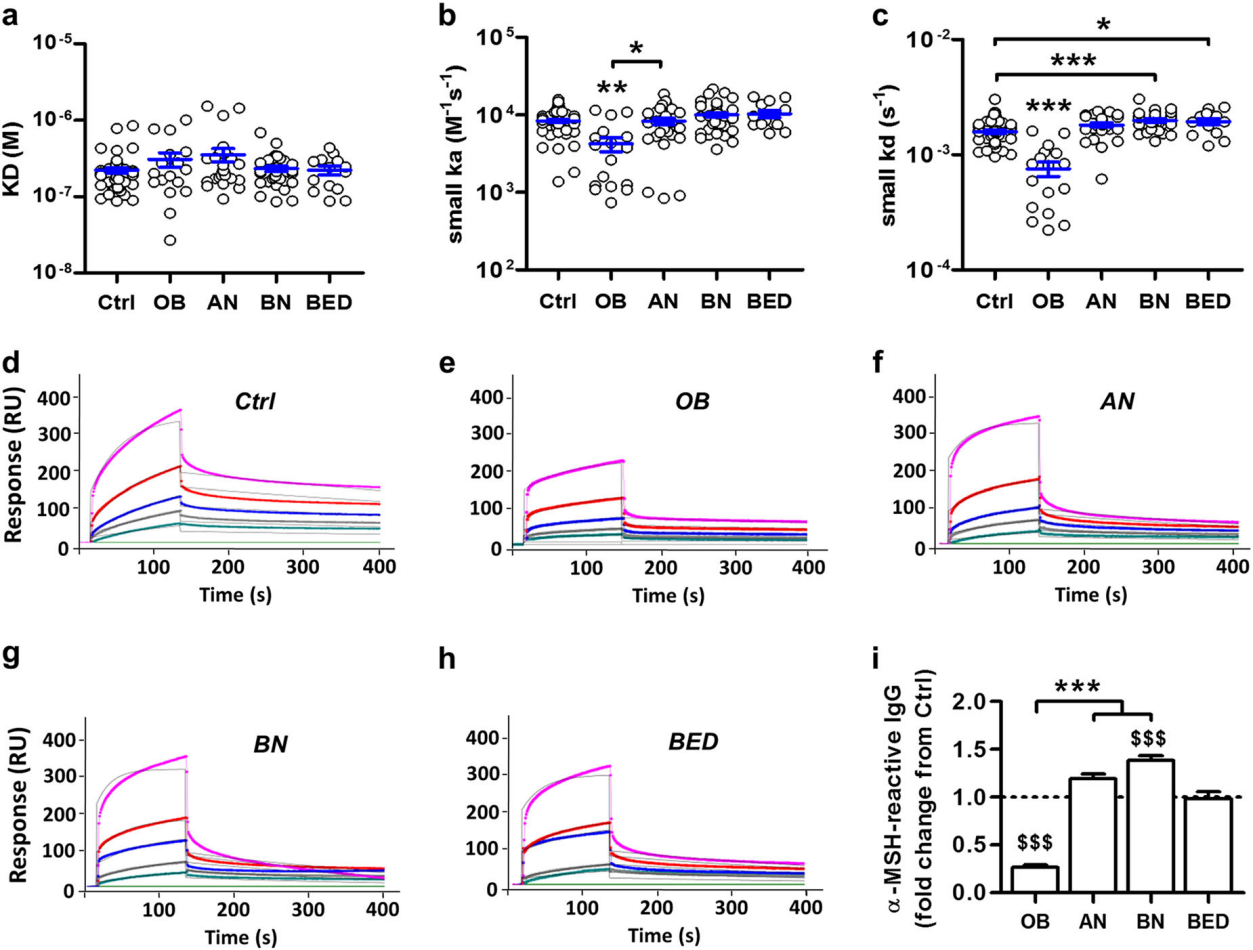
α-Melanocyte-stimulating hormone (α-MSH)-reactive IgG display different affinity kinetic properties in obesity and eating disorders. **a** Dissociation equilibrium constants (KD). **b** Association rates (*ka*). **c** Dissociation rates (kd). **d**–**h** Representative sensorgrams illustrating different affinity kinetics of IgG binding to α-MSH in surface plasmon resonance units (RU) from each study group: **d** Ctrl (*n* = 65), **e** obese (*n* = 17), **f** anorexia nervosa (*n* = 28), **g** bulimia nervosa (*n* = 34), and **h** binge eating disorder (*n* = 14). IgG concentrations, 3360 nM (in pink), 1680 nM (in red), 840 nM (in blue), 420 nM (in grey), 210 nM (in green). Fitting with the Langmuir’s 1:1 model. **i** Relative to controls plasma levels of α-MSH-reactive IgG after their extraction from plasma. Data are means ± s.e.m. Kruskal–Wallis test with Dunns’ post-tests, ***p < 0.001, **p < 0.01, *p < 0.05 (**b**, **c**, **i**) or analysis of variance with Tukey’s post-test, ^$$$^*p* < 0.001 vs. Ctrl (**i**)

To determine whether IgG from OB or ED patients or healthy controls might display different affinity kinetics of α-MSH binding and IC formation, plasma-extracted total IgG were analyzed using SPR (Fig. 1a–h)^24^. We found that the α-MSH affinity (KD) of IgG from all samples was in the micromolar range (Fig. 1a), without significant differences among the groups. However, both association and dissociation rates were decreased in OB patients (Fig. 1b, c). In contrast, dissociation rates were increased in the BN and BED groups (Fig. 1c). A similar tendency was also observed in AN patients (Mann–Whitney test, AN vs. controls, *p* < 0.01).

We also analyzed whether altered α-MSH affinity kinetics of total plasma IgG may exist in animal models of obesity and anorexia. Similar to OB patients, decreased dissociation rates of α-MSH-reactive IgG were found in mice with high-fat-diet (HFD)-induced obesity and in OB Zucker rats (Supplementary Fig. 1). Different from OB patients, IgG from OB *ob/ob* mice and Zucker rats displayed higher affinity (KD) for α-MSH, but this parameter was not affected in HFD-fed OB nor in mice with chronic food restriction or activity-based anorexia (Supplementary Fig. 1).

### Plasma concentrations of α-MSH-reactive IgG are different in OB and ED patients

Plasma concentrations of affinity-purified α-MSH-reactive IgG were analyzed revealing their decrease in OB but increase in BN patients as compared to controls (Fig. 1i). A strong tendency of increased α-MSH-reactive IgG level was also present in the AN group (Mann–Whitney test AN vs. controls, *p* < 0.01).

### α-MSH/IgG IC bind MC4R and is internalized by MC4R-expressing cells differently in OB and ED patients

**Fig. 2:**
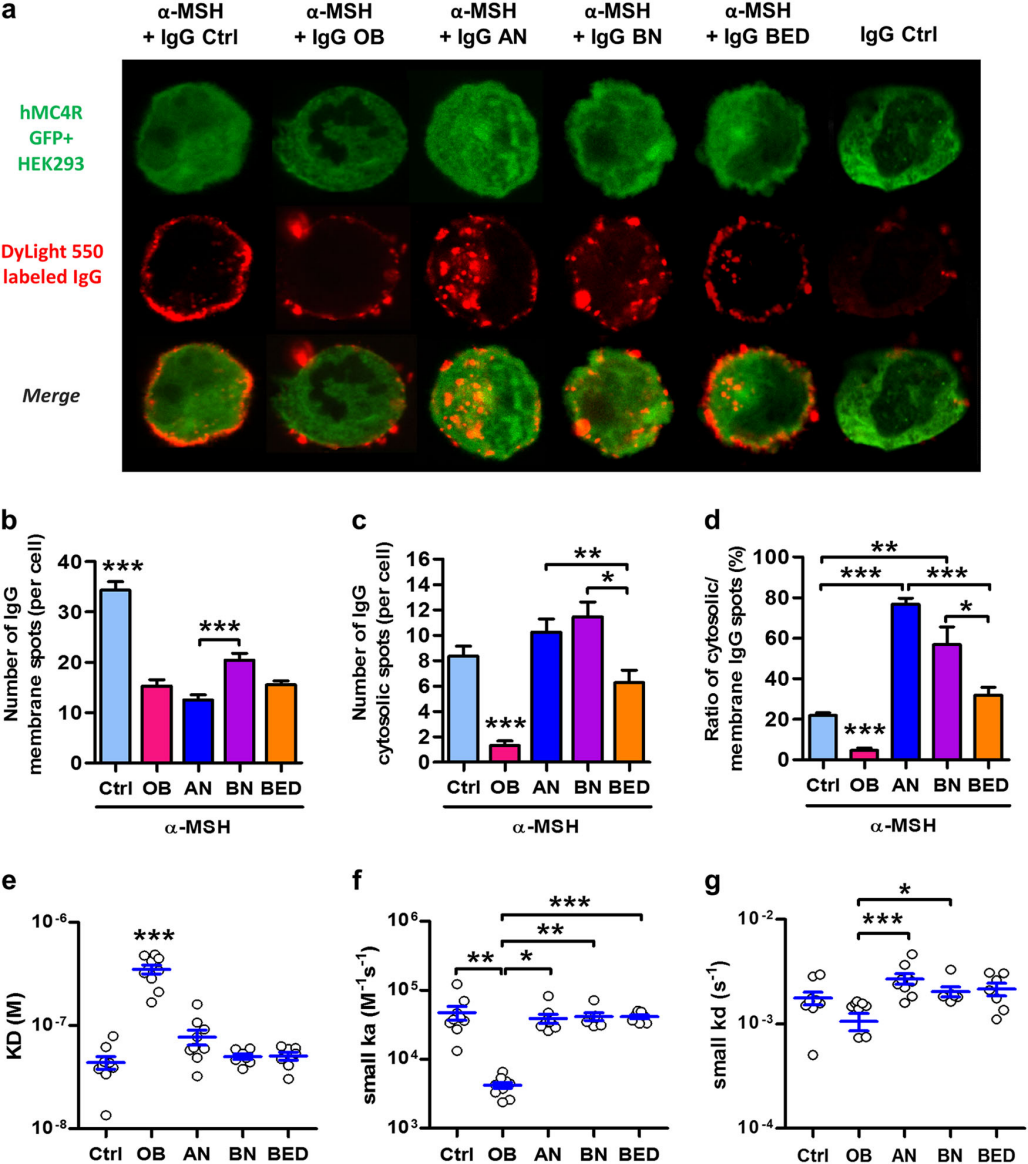
Binding of α-melanocyte-stimulating hormone (α-MSH)/IgG immune complex (IC) to melanocortin 4 receptor (MC4R)-expressing cells is altered in obesity and eating disorders. **a** Representative images of hMC4R GFP+ HEK 293 cells (green) 30 min after application of DyLight 550®-labeled (red) α-MSH affinity-purified IgG from eating disorder (anorexia nervosa, *n* = 9; bulimia nervosa, *n* = 7; binge eating disorder, *n* = 7), obese (*n* = 10), and Ctrl (*n* = 9) preincubated or not with α-MSH. Quantification of DyLight 550®-positive spots in hMC4R+ HEK 293 cells (*n* = 50/group): **b** at the membrane; **c** intracellularly (cytosolic), and **d** ratios of cytosolic/membrane staining. Affinity kinetics properties of α-MSH/IgG IC for hMC4R + HEK 293 cells including **e** dissociation equilibrium constant (KD); **f** association rate (ka), and **g** dissociation rate (kd). Data are means ± s.e.m. Kruskal–Wallis test with Dunns’ post-tests (**b**–**d**, **f**, **g**) or analysis of variance with Tukey’s post-test (**e**), ***p < 0.001, **p < 0.01, *p < 0.05

To determine whether α-MSH-reactive IgG forming IC with α-MSH may bind to the hMC4R in vitro, we used affinity-purified α-MSH-reactive IgG from both patients and controls labeled by DyLight® 550 fluorophore. The binding and uptake by MC4R-expressing cells of IC was analyzed by confocal microscopy. We found that the IC were present both at the cell plasma membrane and within the cytoplasm, likely inside endosomes (Fig. 2a), as previously shown for MC4R internalization upon agonist binding^27,28^. This distribution pattern was specific for α-MSH/IgG IC, as IgG not exposed to α-MSH, but applied alone, displayed only occasional cell membrane association and little or no intracellular uptake (Fig. 2a, most right, illustrated by IgG from the control group). Application of α-MSH/IgG IC to control HEK 293 cells did not result in binding or cellular uptake (data not shown). Further, by quantifying the distribution of IC from patients vs. controls, we found that, while IC based on IgG from controls displayed the strongest cell membrane staining pattern, IC based on IgG from OB patients showed both low cell membrane and the weakest intracellular signals (Fig. 2b, c), indicating reduced binding and uptake of IC. Decreased cell membrane, but not cytoplasmic localization, of IC was also found in all three groups of ED patients as compared to controls (Fig. 2b, c). Within the ED groups, IC from BN had higher membrane location as compared to AN (Fig. 2b), while IC from BED patients had the lowest intracellular uptake (Fig. 2c).

The internalization of the MC4R and its recycling is necessary for the maintenance of receptor functionality^20^. To measure the internalization rate of α-MSH/IgG IC, we analyzed ratios between the intracellular and membrane IgG signals in each individual cell. Opposite changes in such ratios were found for IgG from the OB and AN groups, being lower and higher as compared to controls, respectively (Fig. 2d). An increased ratio of cytosolic uptake was also present in the BN group. In contrast, the low membrane binding and severely reduced internalization of α-MSH IC from OB IgG indicate both their decreased affinity for the MC4R and inhibition of its internalization, which is characteristic for an antagonist binding^27^. In spite of decreased membrane binding of α-MSH/IgG IC in the BED group, no differences in the internalization rate was observed between BED patients and controls (Fig. 2d).

### OB patients display lower affinity of α-MSH/IgG IC binding to MC4R

To further study whether patients display altered α-MSH/IgG IC affinity toward MC4R, we used SPR on hMC4R-expressing HEK 293 cells. We found that, indeed, α-MSH in IC with IgG from OB patients displayed about 10 times lower affinity to MC4R than in IC with IgG from controls (Fig. 2e), which was mainly due to lower association rates (Fig. 2f). No significant differences in affinity kinetics of α-MSH/IgG IC to MC4R were found in the groups of ED patients vs. controls (Fig. 2e–g). Thus α-MSH/IgG IC formed with α-MSH-reactive IgG autoantibodies from OB patients have reduced capacity to bind MC4R.

### α-MSH/IgG IC lower the threshold of MC4R-mediated cAMP activation: a deficient function in OB patients

**Fig. 3:**
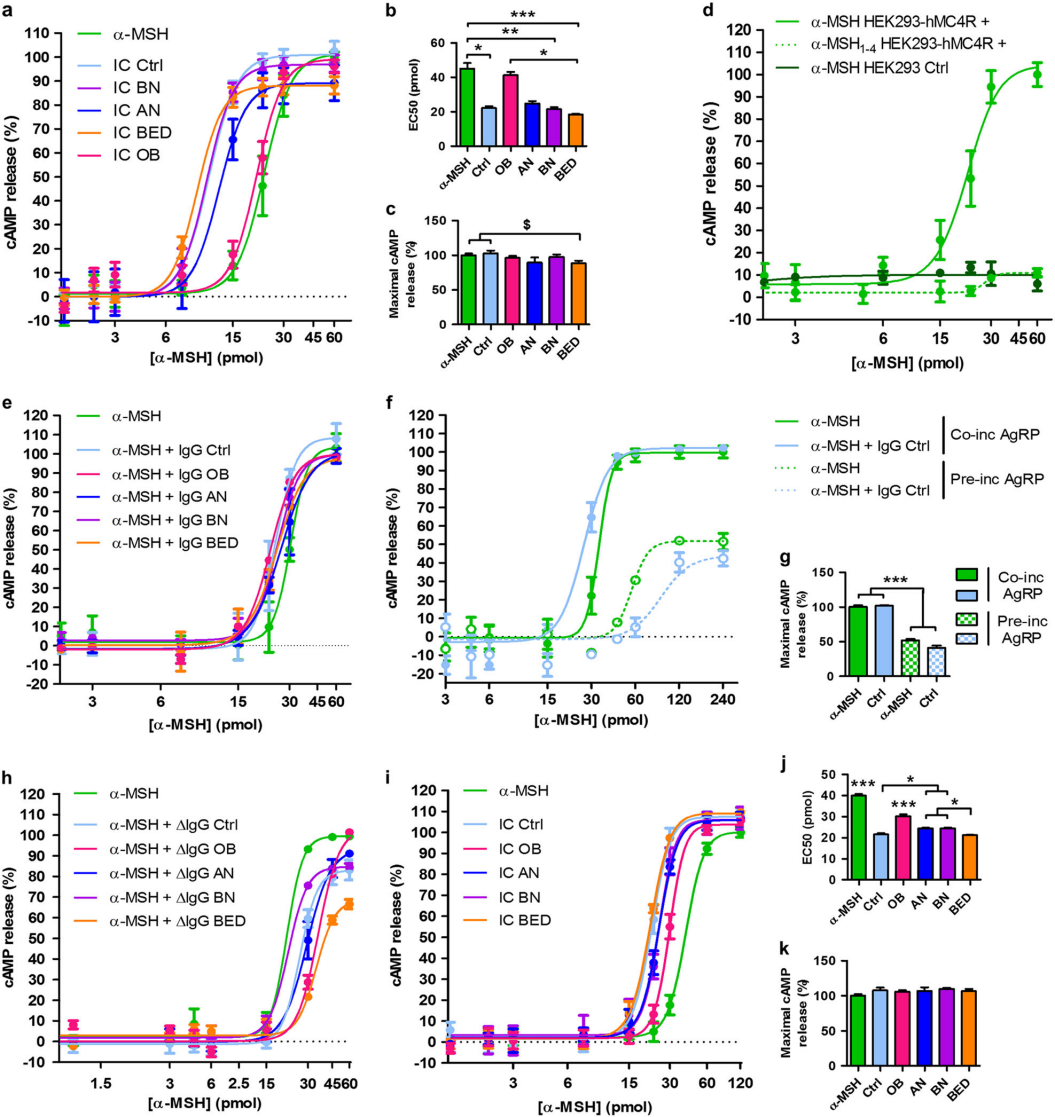
α-Melanocyte-stimulating hormone (α-MSH)/IgG immune complex (IC) lower threshold of α-MSH-induced cyclic adenosine monophosphate (cAMP) release by melanocortin 4 receptor (MC4R)-expressing cells. **a** cAMP dose–response curves to α-MSH alone or α-MSH/IgG IC formed by IgG pooled in patents and control groups and adjusted to α-MSH-reactive IgG plasma levels of controls. cAMP dose–response curves to α-MSH preincubated or not with individual total IgG and corresponding EC50 (**b**) and maximal cAMP production (**c**). **d** Control experiments including cAMP dose–response curves to α-MSH by MC4R-expressing and non-expressing control HEK 293 cells and to α-MSH_1–4_ peptide by MC4R-expressing cells (*n* = 4). **e** cAMP dose-response curves to α-MSH and IgG from patients and controls without their overnight pre-incubation. **f**, cAMP dose–response curves to α-MSH alone or α-MSH/IgG IC co-administered (solid line) with agouti-related protein (AgRP; 100 nM) or added after AgRP preincubation (dotted line—*n* = 2/group) as well as in **g** cAMP maximal response. **h**, **i** cAMP dose–response curves of α-MSH preincubated with **h** purified total IgG from patients and controls depleted for α-MSH-reactive IgG (*n* = 3/group) and **i** affinity-purified α-MSH-reactive IgG (*n* = 6/group); **j** EC_50_ and **k** maximal cAMP production at the plateau. Data are means ± s.e.m. Analysis of variance with Tukey’s post-test (**b**, **c**, **g**, **j**) or Kruskal–Wallis test with Dunns’ post-tests (**m**), ***p < 0.001, **p < 0.01, *p < 0.05; Mann–Whitney test, ^$^*p* < 0.05. **a** α-MSH (*n* = 9), Ctrl, anorexia nervosa (AN), bulimia nervosa (BN), and binge eating disorder (BED; *n* = 6), obese (OB; *n* = 4); **d** HEK 293-hMC4R+ (*n* = 5), HEK 293-CTRL (*n* = 6); **e** α-MSH (*n* = 3), Ctrl, BN, and BED (*n* = 2), AN and OB (*n* = 3)

Next, we determined whether α-MSH/IgG IC may differentially activate MC4R by monitoring cellular cAMP production^29^. Because OB and ED patients had different plasma levels of α-MSH-reactive IgG (Fig. 1i), we studied their effect on MC4R after adjusting total IgG concentrations to normalize the levels of α-MSH-reactive IgG subset relative to the control group. Total IgG were preincubated overnight with α-MSH to form IC and then were applied to hMC4R-expressing HEK 293 cells, and cAMP levels were measured after 15 min. We found a left shift of the cAMP release curve by IC formed by α-MSH and IgG from controls and ED patients with a similar (about 50%) decrease of the EC_50_, as compared to free α-MSH (Fig. 3a, b). However, there was no such potentiation of MC4R signaling by IC formed with IgG from OB patients (Fig. 3a, b). Further, a lower maximal cAMP release was observed with IC formed by IgG from BED patients vs. controls (Fig. 3c). Together, these results show that IC formed by α-MSH-reactive IgG in obesity are associated with reduced MC4R activation, which is in line with decreased MC4R binding and internalization, and low MC4R affinity (Fig. 2).

As negative controls, we did not observe cAMP production after stimulation with the N-terminal tetrapeptide of α-MSH, which does not contain the MC4R pharmacophore (Fig. 3d), nor after applying α-MSH on control HEK 293 cells lacking MC4R (Fig. 3d). Application of IgG alone on either MC4R-expressing or control HEK 293 cells did not result in cAMP stimulation. Further, we observed that IC formation between IgG and α-MSH was necessary for potentiation of MC4R signaling, because the left shift was not present after adding α-MSH and IgG to the cells without the preincubation step (Fig. 3e).

To verify whether α-MSH/IgG IC stimulated cAMP production specifically via MC4R, the MC4R-expressing HEK 293 cells were preincubated with AgRP, a selective MC4R reverse agonist^19^. We found that AgRP antagonized both α-MSH- and α-MSH/IgG IC-induced cAMP production (Fig. 3f, g) confirming MC4R as the receptor responsible for the observed cAMP signal.

Next, to verify whether the MC4R activation threshold-lowering ability of α-MSH/IgG IC was mediated specifically by the α-MSH-reactive IgG, we reproduced the cAMP stimulation experiment after depleting total plasma IgG of the α-MSH-reactive subset by affinity chromatography. We found that, indeed, the mixture of α-MSH with the remaining total IgG, did not produce any left shift of the cAMP release curve (Fig. 3h). In contrast, the use of the affinity-purified α-MSH-reactive IgG fraction preincubated with α-MSH led to a lower threshold of cAMP production (Fig. 3i, j). Moreover, such potentiation of cAMP production was attenuated in the group with α-MSH-reactive IgG from OB patients (Fig. 3i, j), in agreement with the data obtained using total IgG (Fig. 3a, b).

### α-MSH/IgG IC can influence feeding behavior in rodents

**Fig. 4:**
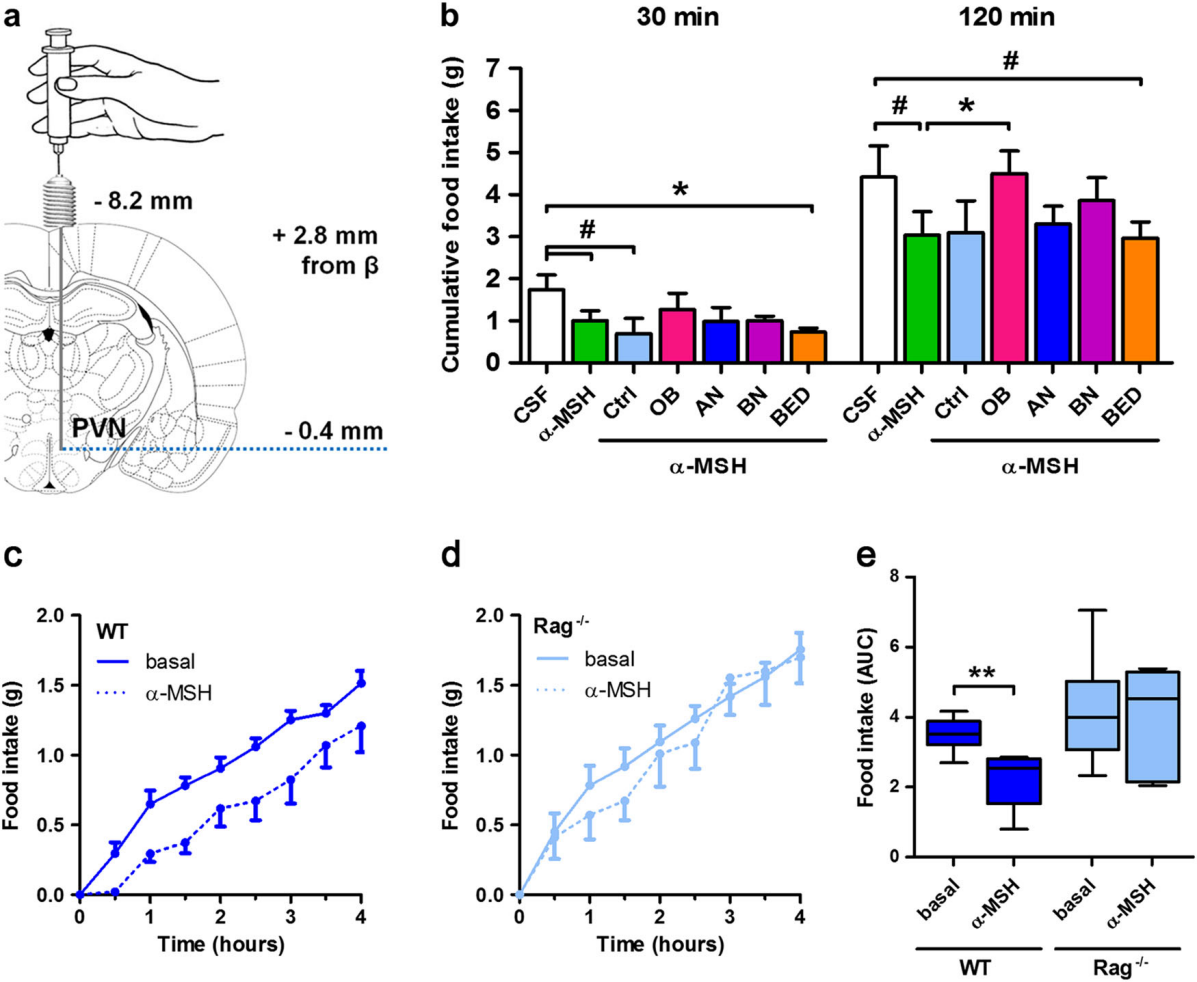
Influence of antibodies on α-melanocyte-stimulating hormone (α-MSH) anorexigenic effects in rodents. **a** Schematic illustration of the injection site in the rat paraventricular nucleus (PVN). **b** Cumulative food intake in rats measured at 30 and 120 min after acute intra-PVN injection of 2 μL of affinity-purified α-MSH-reactive IgG from eating disorder patients (anorexia nervosa, *n* = 5; bulimia nervosa, *n* = 5; binge eating disorder, *n* = 6), obese patients (*n* = 6), and Ctrl (*n* = 4) all preincubated with α-MSH and diluted in artificial cerebrospinal fluid (CSF); control group received CSF only (CSF, *n* = 5). Cumulative food intake in wild-type (WT, *n* = 6) (**c**) and Ig-deficient Rag^−/−^ mice (*n* = 6) (**d**) measured during 4 h after injection of α-MSH (100 µg/kg) (dotted line) as compared to baseline (solid line). Data are means ± s.e.m and expressed as area under the curve (AUC, **e**, right panel). Analysis of variance with Tukey’s post-test (**b**) and Mann–Whitney test (**e**), **p < 0.01, *p < 0.05, ^#^*p* < 0.10

To explore whether α-MSH/IgG IC may influence α-MSH-induced anorexigenic effects in vivo, IgG pools from the patient and control groups were preincubated overnight with α-MSH and injected into the brain of overnight food-deprived rats via preimplanted cannulas targeting the hypothalamic PVN (Fig. 4a), one of the principal central cites of MC4R-mediated anorexigenic action^9^. A significant anorexigenic effect of α-MSH was observed at both 30 and 120 min during refeeding and was not significantly affected by IgG from any group at 30 min, but it was reduced by IgG from OB patients at 120 min (Fig. 4b). The central effect of α-MSH/IgG IC was studied here as an experimental model to reveal potential differences between the IC formed by patients’ and control’ IgG; we recognise that, although α-MSH-reactive IgG have been detected in the CSF of humans^30^, their transport and functional role in the brain needs further studies.

To analyze the peripheral effects of IgG to modulate α-MSH anorexigenic effect, we used transgenic mice lacking Igs due to inactivation of recombination-activating genes (Rag). Rag^−/−^ and wild-type mice were injected IP with α-MSH and their 4-h food intake was measured. We found that α-MSH significantly reduced food intake in wild-type but not in Rag^−/−^ mice (Fig. 4c, d), pointing to the importance of IgG for the physiological anorexigenic effects of peripheral α-MSH. We cannot, however, exclude that lack of other Ig classes in Rag^−/−^ mice may also be involved in the absence of a significant anorexigenic effect of α-MSH.

### α-MSH-reactive IgG display distinct epitope-binding profile in patients with obesity and ED

**Fig. 5:**
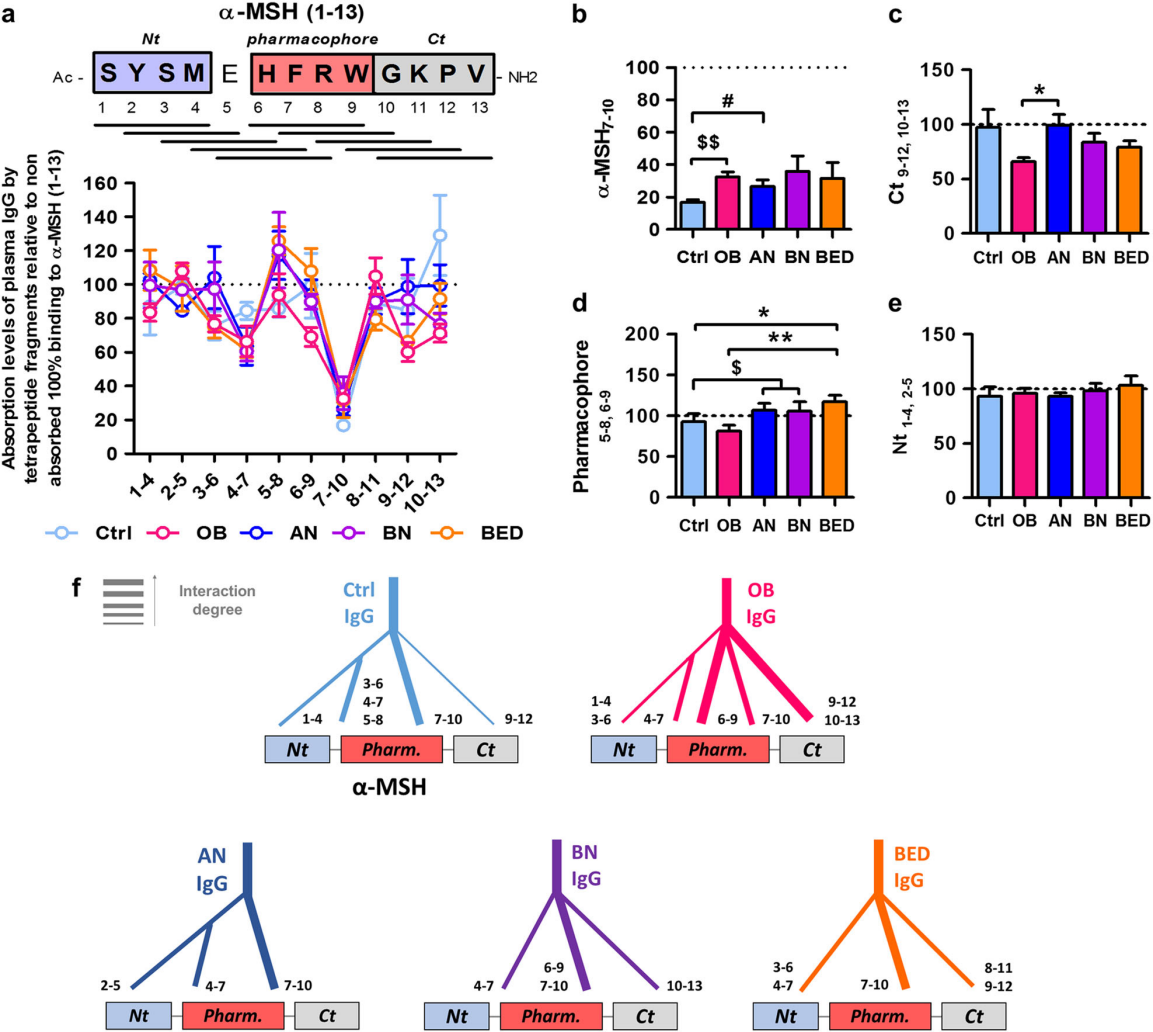
Epitope mapping of IgG for α-melanocyte-stimulating hormone (α-MSH) in patients and controls. **a** Ten tetrapeptide fragments (black lines) overlapping the α-MSH sequence were used for adsorption of total IgG from eating disorder and obese (OB) patients and controls before detection of their immune complex binding by enzyme-linked immunosorbent assay on the entire α-MSH molecule. Adsorption levels of plasma IgG for each fragment was expressed in percentage from non-absorbed total binding to α-MSH (100%). Adsorption levels were compared among the groups for individual tetrapeptides as shown here for: **b** α-MSH_7–10_, the central part which includes 3 amino acids of α-MSH pharmacophore (red box in **a**); **c** the C-terminal (Ct, gray box in **a**), as mean levels of α-MSH_9–12_ and α-MSH_10–13_; **d** the pharmacophore sequence as mean levels of α-MSH_5–8_ and α-MSH_6–9_; and **e** the N-terminal (Nt, blue box in **a**) as mean levels of α-MSH_1–4_ and α-MSH_2–5_. **f** Schematic illustration of α-MSH-binding epitopes in IgG of patients and controls (see also Fig. 6 legend); the degree of adsorption of IgG binding to α-MSH by specific tetrapeptides is shown here by the width of traits toward corresponding parts of the α-MSH sequence. Data are means ± s.e.m. Kruskal–Wallis test, Dunns’ post-test, **p < 0.01; *p < 0.05. Mann–Whitney test, ^$$^*p* < 0.01, ^$^*p* < 0.05, ^#^*p* < 0.10. Ctrl (*n* = 9), OB (*n* = 10), anorexia nervosa (*n* = 9), bulimia nervosa (*n* = 7), binge eating disorder (*n* = 6)

Finally, to get an insight into the functional differences of α-MSH/IgG IC as described above, we analyzed whether α-MSH-reactive IgG from patients and controls might have different α-MSH-binding epitopes. We found that the main IgG epitope in all groups was localized in the central part (amino acids (a.a.) 7–10) of the α-MSH sequence overlapping with the α-MSH pharmacophore^31^ (Fig. 5a, b). However, several alternative epitopes were also present in OB patient IgG, overlapping with the C-terminal (a.a. 9–12 and 10–13, Fig. 5a–c) and with the pharmacophore (a.a. 5–8 and 6–9, Fig. 5a–d). In contrast, reduced binding of the central α-MSH part (a.a. 5–8) was present in IgG from ED patients (Fig. 5a–d). These differences in epitope binding are in line with altered affinity kinetics of IgG as revealed by SPR showing a decrease of dissociation rates in OB and an increase in ED patients (Fig. 1c). No significant differences in IgG binding to the N-terminal of α-MSH were found (Fig. 5a–e). The complete epitope screening data are shown in Supplementary Fig. 2 and are summarized in Fig. 5f. These data suggest that α-MSH-reactive IgG in healthy subjects bind and transport α-MSH mainly through the central part of peptide, making the N- and/or C-terminals available for receptor recognition.

## Discussion

Circulating α-MSH is mainly involved in long-term regulation of appetite and body weight by activation of MC4R and MC3R in the brain and peripheral targets, and plasma levels of α-MSH are inversely associated with BMI^32^. Although not considered as a satiety hormone such as cholecystokinin or peptide YY, both released from the gut upon a meal^33^, postprandial increases in plasma levels of α-MSH have also been reported^34^. The main sources of circulating α-MSH are the brain, pituitary, and the skin^35^.

The present results regarding the affinity kinetics of α-MSH-reactive IgG are consistent with properties of natural autoantibodies in human plasma^36^ and suggest that secreted α-MSH should saturate available α-MSH-reactive IgG and, hence, circulate as an IC. Indeed, α-MSH produced at physiological concentrations and non-protected by a carrier molecule has little chance to reach its biological targets due to rapid degradation by plasma peptidases. No specific α-MSH carrier has been described so far. Our study shows that IC formed by α-MSH and IgG bind and activate MC4R with a lower threshold than α-MSH alone, supporting a role of α-MSH-reactive IgG not only as a natural α-MSH peptide carrier but also as a constitutive allosteric modulator of MC4R binding and internalization.

Such a phenomenon involving an IC with a peptide hormone to regulate receptor signaling has not been yet described for any other peptide. Our results, hence, reveal a novel mechanism that may be of functional significance to peptide signaling in general. In fact, plasma levels of IgG reactive with other peptide hormones, such as corticotropin, oxytocin, and ghrelin, correlate with behavioral modalities, including aggression, anxiety, and depression in humans^37–39^. Whether IC formed by these peptide hormones may similarly regulate signaling by their corresponding receptors remains to be determined.

In this study, we show that both plasma levels and binding properties of α-MSH-reactive IgG are different in OB and ED patients. In particular, lower plasma levels and decreased dissociation rate of α-MSH-reactive IgG found in obesity were associated with reduced binding and internalization of α-MSH/IgG IC and increased threshold for MC4R activation, revealing a molecular mechanism that may contribute to hyperphagia and positive energy balance in OB patients. In contrast, increased internalization rate of α-MSH/IgG IC found in AN and BN patients appears as a distinct feature of these two forms of ED pointing to enhanced activation of the MC4R-mediated satiety signaling possibly via cAMP-independent intracellular pathways. In fact, G-protein-independent signaling by the MC4R has been described and involves the potassium channel Kir 7.1^40^. Other MCR subtypes such a MC3R may also mediate α-MSH/IgG IC central and peripheral actions relevant to the regulation of feeding behavior and underlie complex behavioral and somatic alterations in ED patients^41^. MC3R is involved in regulation of appetite via activation of dopamine neurons in the ventral tegmental area^42^, and it is also abundantly expressed by orexigenic neuropeptide Y neurons of the arcuate nucleus^43^, i.e., directly accessible to circulating macromolecules, including IgG via the fenestrated capillaries of the median eminence^44^.

Further research should also clarify the origin of different plasma levels and properties of α-MSH-reactive autoantibodies in OB and ED patients with a particular attention to gut microbiota known to play a role in the regulation of humoral immunity as well as in control of appetite^45–47^. In fact, caseinolytic protease B (ClpB) produced by *Enterobacteriaceae* was recently identified as an antigen-mimetic of α-MSH^15^. ClpB relevance to ED was further supported by increased plasma concentrations of this bacterial protein correlating with α-MSH-reactive IgG levels in ED patients^16^. However, ClpB was also present in plasma of healthy humans, suggesting that this bacterial protein itself does not have a pathogenic role. Similarities of α-MSH-reactive IgG affinity kinetics between OB patients and rodent models of obesity further support a functional link between α-MSH-reactive IgG and the OB phenotype.

**Fig. 6:**
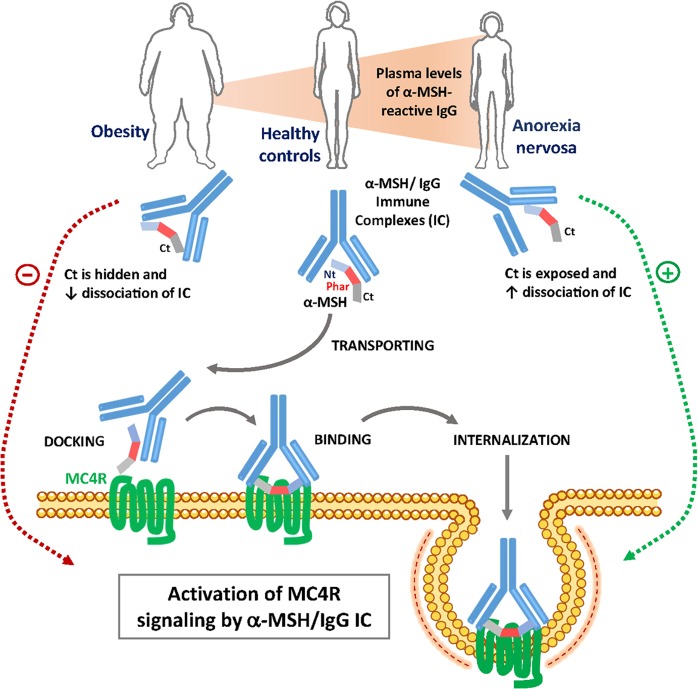
Hypothetical model of α-melanocyte-stimulating hormone (α-MSH)/IgG immune complex (IC) activation of melanocortin 4 receptor (MC4R) in healthy controls and patients with obesity or anorexia nervosa. Plasmatic IgG transport α-MSH by forming IC that bind and activate MC4R. During interaction with the receptor, the C-terminal (Ct) of α-MSH should be presented by IgG enabling IC docking and binding. During binding, α-MSH dissociates from IC and its pharmacophore (Phar) enters the receptor binding pocket, resulting in receptor activation ex. cyclic adenosine monophosphate production. The α-MSH/IgG IC then internalized together with MC4R, resulting in temporal desensitization. As illustrated in Fig. 5f, in healthy subjects IgG bind mainly the central and the N-terminal parts of α-MSH making the C-terminal available for MC4R docking. However, in obese patients the C-terminal is hidden by IgG, preventing α-MSH/IgG IC docking to MC4R. In contrast, in anorexia nervosa (AN) patients, the C-terminal of α-MSH is not bound by IgG, favoring receptor recognition. The changes in α-MSH epitope binding in obesity combined with decreased dissociation of IC and low plasma levels of α-MSH-reactive IgG may cause deficient activation of MC4R, promoting positive energy balance. In contrast, the α-MSH epitope-binding properties of IgG in AN combined with increased dissociation of IC and increased plasma levels of α-MSH-reactive IgG are favorable for more efficient activation of MC4R by α-MSH/IgG IC, resulting in enhanced satiety signaling and negative energy balance

Based on these results, we propose a hypothetical model implicating α-MSH-reactive IgG in MC4R signaling depending on the availability of the C-terminal for receptor recognition (Fig. 6). In fact, the role of both the N- and C-terminals of α-MSH in binding to different MCR subtypes has been shown, with the C-terminal being essential for MC4R recognition^48^. Thus hiding of the C-terminal by IgG in OB patients appears as a negative modulator of α-MSH/IgG IC activation of MC4R. Interestingly, IgG autoantibodies reactive with a.a. 12–13 of the α-MSH C-terminal have also been detected in narcoleptic patients carrying an increased risk of obesity^49^. In contrast to IgG from OB, BN, and BED, IgG from AN patients displayed no binding to the C-terminal, which may facilitate MC4R recognition of α-MSH/IgG IC in AN. The IgG-transporting properties of adrenocorticotropic hormone (ACTH), another melanocortin peptide, have also been recently reported^50^. Of relevance to the present study, the IgG binding to the central part of the ACTH peptide preserved its functional activity to stimulate cortisol secretion.

In conclusion, deficient activation of MC4R by α-MSH/IgG IC may contribute to the pathophysiology of hyperphagic obesity as a result of low level and altered binding properties of α-MSH-reactive IgG in OB patients. In contrast, increased plasma levels of α-MSH-reactive IgG in AN and BN patients combined with altered properties of IC, inducing fast MC4R internalization, may contribute to the behavioral phenotype in these two main forms of ED by enhancing baseline satiety/reduced hunger perception. Thus these data should shed light on the molecular mechanisms of altered appetite in obesity and ED.

## Supplementary information

Supplementary Figure legends.

Supplementary methods.

Supplementary Figure 1.

Supplementary Figure 2.
