# Supplementary material for: Immunoglobulin G modulation of the melanocortin 4 receptor signaling in obesity and eating disorders
Source: Transl Psychiatry. 2019 Feb 12;9:87. doi: 10.1038/s41398-019-0422-9 (PMC6372612; doi:10.1038/s41398-019-0422-9)
Supplement: Supplementary file 1 — Supplementary Figure legends. [file 41398_2019_422_MOESM1_ESM.docx]

**Supplementary figure legends**

**Supplementary figure 1. Affinity kinetics of IgG for α-MSH in animal models of obesity and anorexia.** Association (*ka*) and dissociation (*kd*) rates and dissociation equilibrium constant (*KD*) between α-MSH and IgG purified from plasma of: **a**, obese HFD-fed mice (n=13); **b**, obese *ob/ob* mice (n=7); **c**, obese Zucker rats (n=5) and **d**, activity-based anorexia (ABA, n= 5) and feeding time restricted (FTR, n=6) mice, vs. controls (Ctrl – **a**, n=8; **b**, n=10; **c**, n=6; **d**, n=6). Data are means ± s.e.m. Student t-test (**a**, **c**) or Mann-Whitney test (**b**), $$p<0.01, $p<0.05, #p<0.10; Kruskal-Wallis test, Dunns’ post-test, *p<0.05.

**Supplementary figure 2. α-MSH epitope mapping of IgG.** Adsorption levels were compared among the groups of patients and controls for each α-MSH tetrapeptide fragment relative to non-absorbed IgG binding to the entire α-MSH sequence (100%). **a**, α-MSH_1-4_; **b**, α-MSH_2-5_; **c**, α-MSH_3-6_; **d**, α-MSH_4-7_; **e**, α-MSH_5-8_; **f**, α-MSH_6-9_; **g**, α-MSH_8-11_; **h**, α-MSH_9-12_; **i**, α-MSH_10-13_. Data are means ± s.e.m. One-way ANOVA, Tukey’s post-test, *p<0.05. Student t-test or Mann-Whitney test, $$p<0.01, $p<0.05, #p<0.10.
