# Supplementary material for: Immunoglobulin G modulation of the melanocortin 4 receptor signaling in obesity and eating disorders
Source: Transl Psychiatry. 2019 Feb 12;9:87. doi: 10.1038/s41398-019-0422-9 (PMC6372612; doi:10.1038/s41398-019-0422-9)
Supplement: Supplementary file 2 — Supplementary methods. [file 41398_2019_422_MOESM2_ESM.docx]

**Supplementary data**

**Materials and Methods - extended**

*IgG purification*

Total IgG were purified from plasma using MelonGel® Purification Kit (LifeTechnologies, Carlsbad, CA, USA). Briefly, mini spin columns were loaded with 500 µL of Melon Gel purification support and centrifuged 30 s at 5000 rpm. After two washes with Melon Gel purification buffer under the same centrifugation conditions, 500 µL of plasmas (diluted 1:4 vol. in purification buffer) were incubated in the columns 5 min at room temperature on a roller mixer (Stuart SRT6). To collect purified IgG, mini spin columns were centrifuged 30 s at 5000 rpm and samples were lyophilized (Bioblock Scientific Christ alpha 1-2) for 48 h, re-suspended in phosphate-buffered saline (PBS) or HBS-EP buffer (GE Healthcare) for affinity kinetics analysis and other experiments and conserved at -80 °C. IgG concentrations were evaluated using NanoDrop 2000C (ThermoFisher Scientific, Waltham, MA, USA) with PBS or HBS-EP buffer as blanks. The same stock solution of IgG from each patient and control was used throughout the study.

*Affinity kinetics of IgG for α-MSH*

Affinity kinetics of plasma extracted IgG for α-MSH were analyzed by biospecific interaction assay (BIA) based on surface plasmon resonance (SPR) phenomenon on a BIAcore 1000 instrument (GE Healthcare), according to a previously published protocol ([Legrand et al., 2014](#_ENREF_27)). α-MSH peptide (Bachem AG, Bubendorf, Switzerland) was diluted in 10 mM sodium acetate buffer, pH 5.0 (GE Healthcare, final concentration = 0.5 mg/ml) and was covalently coupled on the CM5 sensor chip (GE Healthcare). A multi-cycle affinity kinetic analysis method was run with five serial dilutions of purified IgG: 3360, 1680, 840, 420, 210 nM in HBS-EP buffer (0.01 M HEPES pH 7.4, 0.15 M NaCl, 3 mM EDTA and 0.005% surfactant P20, GE Healthcare). Two HBS-EP buffer-only blanks were also performed at the beginning and the end of each cycle. A cycle included 120 s of analyte injection and 300 s of dissociation with a 30 µl/min flow speed at 25 °C. Between sample injections, the binding surface was regenerated with 10 mM NaOH resulting in a return of baseline level of the sensorgram. Affinity kinetic data were analyzed using BiaEvaluation 4.1.1 program (GE Healthcare) and fitted with the Langmuir's 1:1 model after blank values subtraction.

For the affinity kinetics assay of IgG for α-MSH in animal models of obesity and anorexia we used the same protocol for IgG purification and SPR technique as described above. Plasma extracted IgG were available from our previous published studies including mice with HFD-induced obesity ([François et al., 2016](#_ENREF_15)), *ob/ob* mice ([Takagi et al., 2013](#_ENREF_36)) and mice with chronic feeding time restriction (FTR) and activity-based anorexia (ABA) ([Legrand et al., 2016](#_ENREF_26))*.* Male obese fa/fa Zucker rats (550-600g; n=6) and lean *Fa/Fa* Zucker rats (450-500g, Ctrl; n=5) were also used.

*Affinity kinetics of α-MSH/anti-α-MSH IgG immune complexes for the MC4R*

Affinity kinetics of α-MSH/IgG immune complexes for the MC4R, was analyzed using SPR on whole cells. Cell line of human embryonic kidney (HEK) 293 cells that stably expressing human MC4R (hMC4R) was generated using a lentiviral transduction technology and purchased from AMS bio (AMS Biotechnology, Abingdon, UK)**.** High expression of MC4R mRNA in transfected cells was validated by RT-PCR. After unfreezing, HEK293 cells were cultured in 250 ml tissue culture flasks (BD-Falcon, Franklin Lakes, NJ, USA) using supplemented Dubecco's modified Eagle medium (DMEM; 4.5 g/L glucose, 2 mM L-glutamine, 10 % fetal bovine serum, 0.1 mM non-essential amino acids solution, 1% penicillin-streptavidin). Cultured cells were maintained in humidified incubator (37.5 °C, 5% CO_2_). The day of experiment, cultured cells were treated with 0.25% trypsin-EDTA (Sigma-Aldrich) and cell pellets were re-suspended in HBS-EP buffer (0.01 M HEPES pH 7.4, 0.15 M NaCl, 3 mM EDTA and 0.005% surfactant P20, GE Healthcare). Cells were diluted in 10 mM sodium acetate buffer, pH 5.0 (GE Healthcare, final concentration=0.5 mg/ml) and were covalently coupled on the CM5 sensor chip (GE Healthcare). A multi-cycle affinity kinetic analysis method was run with five serial dilutions of purified IgG preincubated with 1 µM α-MSH for 2h at RT: 3360, 1680, 840, 420, 210 nM in HBS-EP buffer. Two HBS-EP buffer-only blanks were also performed at the beginning and the end of each cycle. A cycle included 120 s of IgG injection and 300 s of dissociation with a 30 µl/min flow speed at 25 °C. Between sample injections, the binding surface was regenerated with 10 mM NaOH resulting in a return of baseline level of the sensorgram. Affinity kinetic data were analyzed using BiaEvaluation 4.1.1 program (GE Healthcare) and fitted with the Langmuir's 1:1 model after blank values subtraction.

*α-MSH-reactive IgG purification*

Pooled total IgG purified from patient’s plasma (OB, n=10; AN, n=10; BN, n=8; BED, n=7) and controls (n=10) were further purified using α-MSH-linked UltraLink® Biosupport (Thermo Fisher Scientific, Waltham, MA, USA). Briefly, α-MSH peptide (5 mg) and UltraLink® beads were diluted in a sodium citrate 0.6 M, MOPS 0.1 M buffer and centrifuged during 10 min at 1200 g. After 2 h incubation on a roller mixer at RT, ethanolamine 3 M (10:1 vol. beads) was added and incubated for 2.5 h in the same condition. After washing with PBS under centrifugation 10 min at 1200 g, α-MSH-linked beads were incubated with 1 M NaCL during 15 min at RT to stabilize the covalent link. Then beads were incubated with 250 to 500 µL of purified IgG for 2 h at 4 °C and washed twice with PBS. Elution was made with a 0.1 M glycine, 2% acetic acid solution (pH 2.2) under centrifugation. Finally, samples were lyophilized (Bioblock) for 24 h, suspended in PBS and conserved at -30 °C. Concentrations of purified anti-α-MSH IgG were measured using NanoDrop 2000C (Thermo Fisher Scientific).

*Confocal microscopy*

To characterize the physical interaction between α-MSH-reactive IgG and MC4R we used cell line of HEK 293 cells stably expressing hMC4R (AMS Biotechnology, Abingdon, UK)**.** After unfreezing and pre-culture as described above, HEK293 cells were cultured in glass bottom Petri dishes (MatTek, ≈ 250000 cells/dish) supplemented with DMEM in humidified cell culture incubator for 24 h. Purified anti-α-MSH IgG of each patient and control groups (2 mg/mL in PBS) were conjugated with DyLight® 550 using Lightning-Link® rapid conjugation kit (Innova Biosciences, Cambridge, UK) and then incubated with 200 mM of α-MSH peptide (Bachem) overnight at 4 °C. The day of experiment, hMC4R-GFP+ HEK 293 cell medium was removed and replaced by 100 µL of pre-incubated α-MSH/DyLight® 550 labeled anti-α-MSH IgG (0.5 mg/mL in PBS). As a control, DyLight® 550-labeled α-MSH IgG from control subjects (and also AN patients, data not shown) were added on MC4R-GFP+ cultured HEK 293 cells without pre-incubation with α-MSH peptide. After incubation during 30 min at 37 °C, cells were washed twice with cold PBS and fixed in a solution of 4 % paraformaldehyde (PFA) in a 0.16 M phosphate buffer (pH 6.9 at 37 °C) for 15 min. After three washes, cells were conserved in PBS at 4 °C and observed on a reversed confocal laser scanning microscope TCS SP2 AOBS-DMIRE2 (Leica, Wetzlar, Germany). Fluorescence of GFP was excited using a 488 m-Ar/Kr laser, detected with a band pass filter of 500 to 550 nm. DyLight® 550 was excited at 561 nm and detected with a 570-620 nm band pass filter. Pictures were taken with x45 and x60 oil-immersion objectives at the optical planes where cells nuclei were the most voluminous. Data were analyzed using Leica Confocal Software (Leica) and DyLight® 550-positive spots of GFP+ cells were visually quantified (n*=*50 for each condition).

*In vitro cAMP assay*

Stable cultured HEK 293 cell line were treated with 0.25 % trypsin-EDTA, cell pellet was re-suspended in PBS and filed in a non-culture treated white 96-microwell plate (Nunc, ≈5000 cells/10 µL/well). The cyclic adenosine monophosphate (cAMP) production by MC4-R expressing HEK 293 cells was measured using the bioluminescence assay cAMP-Glo^TM^ Max assay kit (Promega, Madison, WI, USA) according to the manufacturer's instructions. Briefly, cells were incubated 15 min at 23°C with different dilutions of α-MSH peptide (from 1 to 60 pmol, Bachem). Serial dilutions of cAMP standard (provided by the kit) were assayed on the same microplate. Lysis buffer and cAMP detection solution were added to each well and cells were homogenized by agitation and centrifugation during 2 min at 1000 rpm. Then, the plates were incubated for 20 min at 23°C in a dry incubator. Kinase-Glo reagent substrate was added in each well and the luminescence was read with a bioluminescence detection instrument (Safas Spectrometer, Monaco). The same experiments were also performed on HEK 293 control cells which were not transfected with the MC4R resulting in no significant α-MSH-induced cAMP production (2.73±1.43 nM at 60 pmol of α-MSH). As another negative control, a truncated N-terminal α-MSH tetrapeptide (α-MSH_1-4_, n=4) that didn’t contain the α-MSH, pharmacophore necessary for MC4R activation, was used in the same dilution range^23^.

To test the effect of IC with α-MSH-reactive IgG on MC4-R signaling, the range of α-MSH peptide was pre-incubated the day before the experiment with pooled purified IgG (1/14.5 in PBS according to the Ctrl IgG affinity purification yield for α-MSH) from Ctrl (n=6), OB (n=4) and ED (n=6) patients or with PBS (n=9) overnight at 4°C.

The range of α-MSH peptide and pooled purified IgG from Ctrl (n=2), OB (n=3) and ED (n=2) patients or with PBS (n=3) were also co-administrated on MC4R+ HEK293 cells without pre-incubation.

To confirm the specificity of MC4-R dependent cAMP release, MC4R+ HEK 293 cells were finally pre-incubated with specific MC4-R reverse agonist AgRP. Briefly, MC4R+ HEK 293 cells were cultured in a 96-well plate in supplemented DMEM for 24h. The day of experiment, cells were incubated with AgRP (100 nM) for 45 min and washed three times with PBS (37°C) before proceeding to cAMP release measurements with α-MSH alone or pre-incubated with purified IgG from Ctrl (n=2/ group). As a control, AgRP was also added on cells with α-MSH alone or pre-incubated with purified IgG from Ctrl (n=2/group).

Finally, to confirm the specificity of anti-α-MSH IgG effects on MC4R signaling, the range of α-MSH was pre-incubated overnight at 4°C with IgG depleted from α-MSH-reactive IgG obtained in the purification process described above (n=3/group) or with affinity purified α-MSH-reactive IgG (n=6/group).

*α-MSH -reactive IgG epitope mapping*

IgG autoAbs epitope mapping vs. α-MSH peptide was performed using an ELISA technique following adsorption of IgG by α-MSH fragments. Total IgG from obese patients were diluted at 1:400 in sample buffer (PBS with 0.02% NaN_3_) and IgG from other groups were normalized to the concentration of α-MSH-reactive IgG in the obese group and incubated overnight at 4°C with each of 10 different α-MSH tetrapeptide fragments (Bachem – 1:2 vol.). As a control, IgG samples were also incubated with the sample buffer only. α-MSH peptide (Bachem) was coated at 2 µg/mL in 0.5 M carbonate-bicarbonate buffer pH 9.6 containing 0.02% NaN_3_ on 96 wells plates overnight at 4 °C. Then coating solution was discarded and plates were washed three times in PBS with 0.05% Tween 20 (wash buffer) and 100 µL of IgG preadsorbed samples were incubated overnight at 4 °C. Each IgG sample was assayed in duplicate. The third day of experiment, sample solution was discarded and plates were washed 3 times. The detection alkaline phosphatase (AP)-conjugated anti-human IgG antibody (Sigma) was diluted in sample buffer (1:2000) and incubated (100 µL/well) 3 h at RT. After 3 washes, the antibody solution was replaced by the AP substrate (100 µL/well) for 40 min at RT. Finally, the reaction was stopped (3N NaOH – 50 µL/well) and the optical density (OD) was measured at 405 nm with an ELISA microplate reader Metertech 960 (Bioblock). Adsorption levels were analyzed first by subtracting blanks OD values from each sample reading and then by calculating the relative change of pre-incubated OD for each tetrapeptide as compared to the non-adsorbed OD values taken as 100%.

*Brain injections of α-MSH IgG IC in rats*

Animal care and experimentation were in accordance with guidelines established by the National Institutes of Health, French and European Community regulations (Official Journal of the European Community L 358, 18/12/1986). Upon arrival into a specialized air-conditioned animal facility, male Sprague-Dawley rats (200-220 g – n=31) purchased from Janvier Labs (L’Arbresle, France) were kept in holding cages (2 rats per cage) in environmental conditions (23 ± 1 °C, 12:12 h light-dark cycle) with standard pellet rodent chow (RM1 diet, SDS, UK) and water available ad libitum. After 7-8 days of acclimatization, rats were anesthetized by an intraperitoneal injection of a ketamine (75 mg/kg, Virbac, Carros, France) / xylazine (5 mg/kg, Bayer, Leverkusen, France) solution (3:1 vol., 0.1 mL/100 g) and were stereotaxically implanted with a 9 mm internal guide cannula for acute injections (PlasticsOne, Roanoke, VA, USA) into the hypothalamic PVN. The coordinates were - 0.4 mm lateral to the midline, + 2.8 mm posterior to the Bregma and - 8.2 mm below the surface of the skull. To maintain the cannula, three screws were fixed on the skull and mounted with resin (Dentalon plus, Heraeus Kulzer, Hanau, Germany). To protect the guide cannula from dust, a dummy cannula was inserted into the guide after operation. Upon awakening, rats were individually maintained in metabolic cages (Techniplast, Louviers, France) for 5-6 days. RM1 pelleted chow was replaced by the same grounded one in order to allow food intake measurement. Animals that did not return to their pre-operative body weight 5 days after operation were excluded from the experiment. At day 6, 12 hour fasted rats were injected at the beginning of the dark phase using a 5 µl Hamilton syringe (Hamilton Company, Reno, NV, USA). Each animal received 2 µL of α-MSH peptide (2.5 mg/mL) alone (n=11) or pre-incubated with 0,82 mg/mL of IgG from AN (n=5), BN (n=5), BED (n=6), OB (n=6) or Ctrl (n=4) subjects in artificial cerebrospinal fluid (aCSF; PhyMep, Paris, France). A control group (n=5) was injected with 2 µL of aCSF. Food intake was measured 30 min and 2 h after injection. Drinking water was always available ad libitum.

*Intraperitoneal administration of α-MSH in immunodeficient Rag-/- mice*

Upon arrival into the animal facility, male C57Bl/6 mice (7 weeks – n=6) purchased from Janvier Labs and Rag^-/-^ mice (generated by the Inserm UMR905 laboratory in a specific-pathogen-free area, n=6) were housed in standard cages (3 mice per cage) in environmental conditions (23 ± 1 °C, 12:12 h light-dark cycle) with standard pellet rodent chow and water available ad libitum. After 7 days of acclimatization, animals were individually housed in BioDaq® cages (Research Diets Inc., New Brunswick, NJ, USA) that provide automatic food intake measurements. During 7 days, the basal food intake of each animal was measured during the first 4 hours of the dark phase. At day 8, intraperitoneal injections of a-MSH (100 µg/kg, Bachem) were performed at the beginning of the dark phase. Individual food intake after injection was compared to the basal food intake of the pre-treatment session in the same day period. Finally, mice were euthanized by lethal administration of ketamine/xylazine solution.

*Statistical analysis*

Data were analyzed and graphs were plotted using the GraphPad Prism 5.02 (GraphPad Software Inc., San Diego, CA, USA). Group differences were compared by the analysis of variance (ANOVA) with Tukey’s post-tests or Kruskal-Wallis test followed by Dunn’s post-tests according to normality results evaluated by the Kolmogorov-Smirnov test. When mentioned, individual differences were analyzed using Student’s *t*-test or Mann-Whitney’s test when the data distribution was respectively Gaussian or not. Results of affinity kinetics were analyzed by Chi^2^ test from BiaEvaluation program. The cAMP production results were fitted using a non-linear regression model (log(α-MSH) vs. normalized cAMP response from zero baseline) which equation was *y=100/(1+10^((logEC50-x)*hillslope))*. All results were expressed as mean ± standard error of mean (s.e.m.), unless specified; and for all tests, *p*<0.05 was considered statistically significant.
