# Supplementary figures and images for: Immunoglobulin G modulation of the melanocortin 4 receptor signaling in obesity and eating disorders
Source: Transl Psychiatry. 2019 Feb 12;9:87. doi: 10.1038/s41398-019-0422-9 (PMC6372612; doi:10.1038/s41398-019-0422-9)

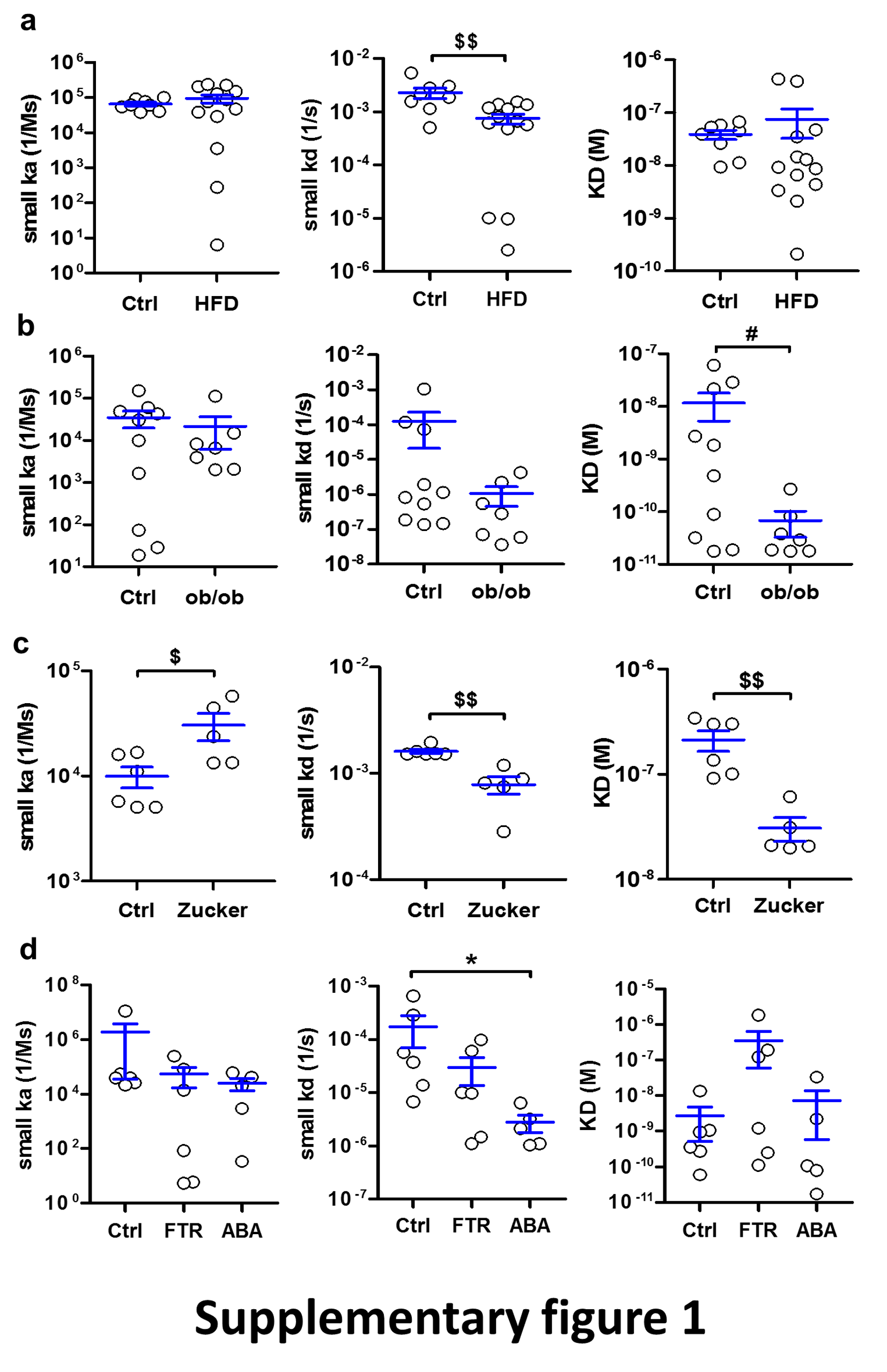

Supplement: Supplementary file 3 — Supplementary Figure 1. [file 41398_2019_422_MOESM3_ESM.tif]

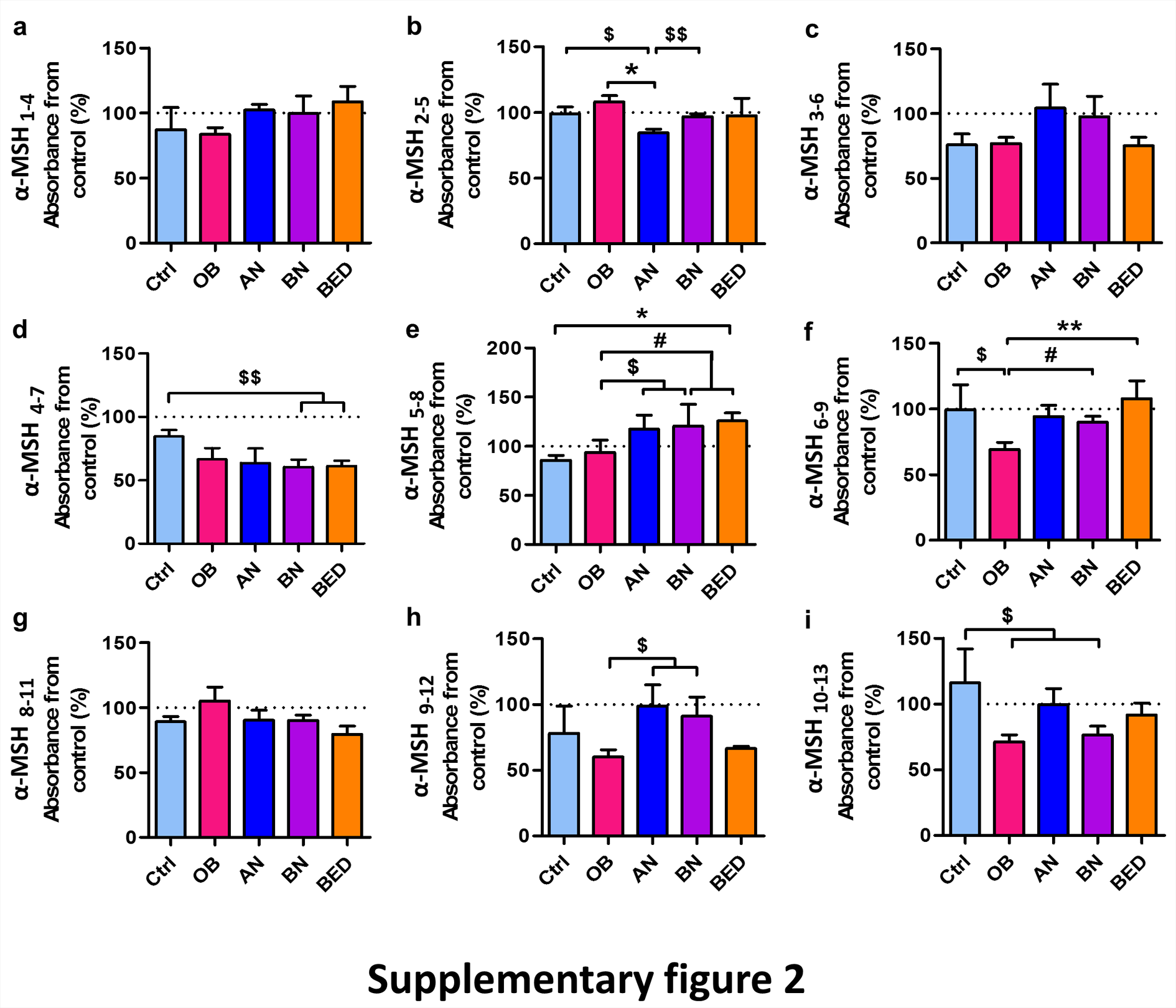

Supplement: Supplementary file 4 — Supplementary Figure 2. [file 41398_2019_422_MOESM4_ESM.tif]
